# A Tuboovarian Abscess Associated with a Ruptured Spleen

**DOI:** 10.1155/2016/8796281

**Published:** 2016-01-24

**Authors:** Jennifer S. Li, Johnathan Michael Sheele

**Affiliations:** Department of Emergency Medicine, University Hospitals Case Medical Center and Case Western Reserve University, 11100 Euclid Avenue, B-517, Cleveland, OH 44139, USA

## Abstract

We report the first case of a tuboovarian abscess complicated by a ruptured spleen. Our patient was a 27-year-old female with human immunodeficiency virus (HIV) who presented to the emergency department (ED) with complaints of urinary symptoms and diarrhea. After being diagnosed with a tuboovarian abscess (TOA), she received antibiotics and was admitted to the gynecology service. Shortly thereafter she developed hemorrhagic shock, necessitating a splenectomy and salpingooophorectomy from a ruptured spleen.

## 1. Introduction

A TOA is an inflammatory mass involving the fallopian tube and ovary. It is one of the most common causes of a pelvic mass in reproductive age women, with approximately 66,000 cases in the United States annually [1]. Risk factors for TOA include multiple sexual partners, not using contraceptives, and a history of pelvic inflammatory disease (PID) [2]. Up to 46% of patients with TOAs report a prior history of PID, and TOA is a complication of 15% of patients with PID [3, 4].

TOA can be a potentially life-threatening condition leading to sepsis or a ruptured abscess, with 15% of cases requiring aggressive medical and surgical intervention [1]. In postmenopausal women, TOAs have been associated with a high risk of malignancy [5]. Other possible complications of tuboovarian abscesses include infertility, ectopic pregnancy, pelvic thrombophlebitis, chronic pelvic pain, and ovarian vein thrombosis [1].

We report a case of a surgically confirmed tuboovarian abscess in a HIV-positive female with an intrauterine device (IUD) who presented to the ED with urinary symptoms and diarrhea. A spontaneously ruptured spleen leading to hemorrhagic shock and an emergent splenectomy and salpingooophorectomy complicated her inpatient clinical course. A ruptured spleen previously has not been reported as a complication of a TOA.

## 2. Case Presentation

**Figure 1:**
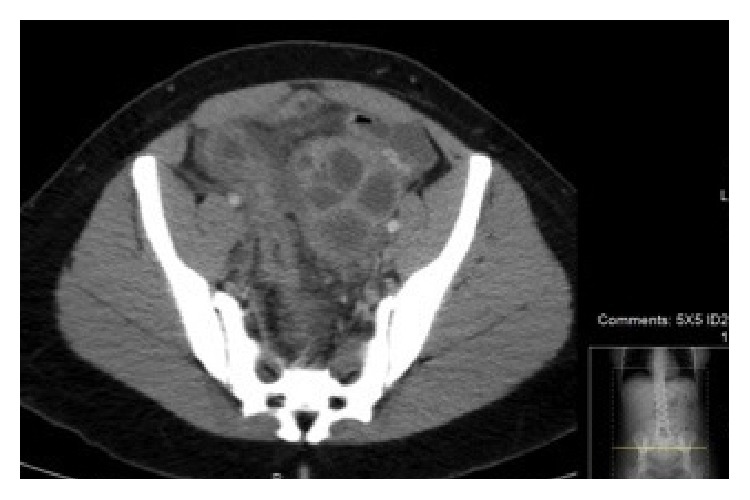
CT scan showing a large multicystic left ovarian mass measuring 6 × 9 cm.

We present a case of a G1P1001 27-year-old female with HIV on highly active antiretroviral therapy (HAART) with an unknown CD4 count and an undetectable viral load who had a single episode of gonorrhea treated nine years ago. She had a Mirena intrauterine device (IUD) placed several years ago and was in a year-long monogamous relationship with a female partner. The patient presented for her first ED visit with complaints of dysuria, abdominal pain, and constipation. She was diagnosed with a urinary tract infection and prescribed seven days of double-strength trimethoprim/sulfamethoxazole before being discharged home. She presented to our ED one month later for her second visit with complaints of dysuria, urinary frequency, and diarrhea. She reported nonbloody diarrhea and intermittent, bilateral abdominal, and lower back pains. In the ED, the patient's temperature was 38.7°C. Her abdomen was diffusely tender with no guarding or CVA tenderness. Pelvic exam revealed white vaginal discharge but no cervical motion or adnexal tenderness. Her urine pregnancy test was negative. Her urinalysis showed 1+ leukocyte esterase but no nitrites, and microscopic urinalysis showed 3 white blood cells, 2 squamous cells, and 4+ bacteria. The patient had a leukocytosis of 16.1 × 10^3^, but her hepatic function panel and lipase were normal. Wet prep was negative for* Trichomonas vaginalis*, clue cells, or yeast. A computed tomography (CT) scan of the abdomen and pelvis with intravenous (IV) contrast showed a large, multicystic left ovarian mass measuring approximately 6 × 9 cm (Figure 1).

The patient was treated with IV doxycycline and ampicillin-sulbactam for a TOA and admitted on the gynecology service. During her hospitalization, she was managed with IV cefoxitin (2 grams every 6 hours), doxycycline (100 mg twice a day), and metronidazole (500 mg twice a day). Gonorrhea, chlamydia, hepatitis B, and syphilis tests were negative. After an infectious diseases consult she was discharged from hospital day #7 with a two-week course of oral ciprofloxacin and amoxicillin-clavulanic.

**Figure 2:**
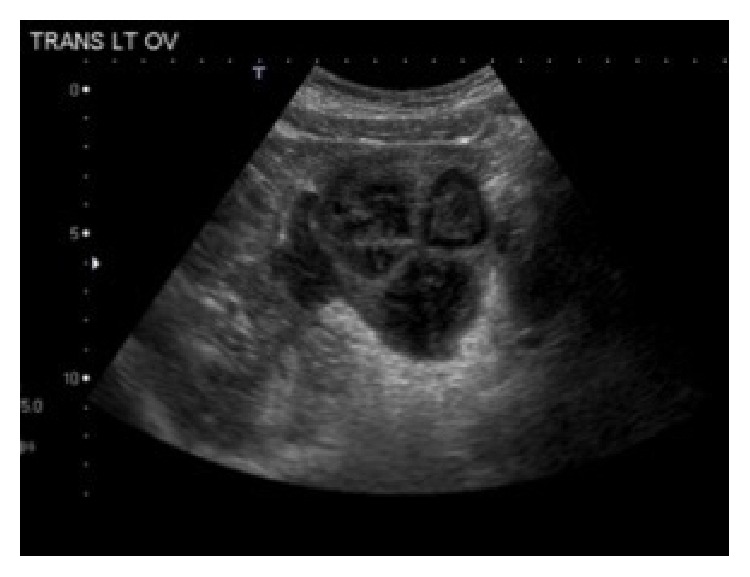
Ultrasound image showing large, complex cystic mass in the left adnexa measuring 5.9 × 8.4 × 5.5 cm in dimensions.

One month later the patient returned for a third ED visit complaining of abdominal pain and chills. The patient was afebrile with a white blood cell count of 10.5 × 10^3^. Physical exam showed diffuse abdominal tenderness, guarding, and both cervical and bilateral adnexal tenderness. Wet mount was positive for* Trichomonas vaginalis* and clue cells. Pelvic ultrasound showed a complex cystic mass in the left adnexa measuring 5.9 × 8.4 × 5.5 cm (Figure 2) suggesting a persistent, left-sided TOA.

The patient was treated with intramuscular ceftriaxone (250 mg), IV doxycycline, and metronidazole in the ED and readmitted to the gynecology service where she was started on IV ampicillin (1 gram every 6 hours), clindamycin (900 mg every 8 hours), and gentamicin (400 mg every 24 hours). On hospital day #2, she underwent ultrasound (US) and computed tomography- (CT-) guided transvaginal drainage of her left tuboovarian abscess by interventional radiology (IR), and 35 mL of frank pus was drained. The gram stain showed 4+ mixed bacteria with no predominant organism. Cultures from the drainage showed no growth aerobically or anaerobically.

On hospital day #4, the patient acutely decompensated with tachycardia, hypotension, anuria, abdominal pain, and a drop in her hemoglobin from 12 to 6 g/dL. She received an emergent exploratory laparotomy and was found to have massive hemoperitoneum due to a ruptured spleen. There was no evidence of short gastric vessels to the spleen. The patient required five units of packed red blood cells, four units of fresh frozen plasma, a splenectomy, and a left salpingooophorectomy. She was discharged home on postop day #6 with a two-week course of oral doxycycline and metronidazole.

The pathology report of the spleen noted acute inflammation involving the splenic capsule and focal evidence of remote hemorrhage and possible splenic tear in the hilar region. The operative report also noted inflammation in the upper abdomen. It is possible that infectious material from the TOA extended superiorly, causing adhesion with the spleen leading to the splenic rupture. There were no complications reported by IR during the US- and CT-guided drainage of the TOA.

## 3. Discussion

Wandering spleen is a rare condition involving laxity of the splenic ligament. It is found in less than 0.25% of the patients who require splenectomy [6]. One-third of patients with wandering spleen are children under the age of 10 [6]. In adults, a wandering spleen usually presents between the ages of 20 and 40 years and is ten times more common in females than males. Wandering spleen can be detected on ultrasound or CT, but there were no radiographic reports of the condition on her previous imaging studies [7]. The definitive management for a wandering spleen is splenectomy.

TOAs are most commonly polymicrobial aerobic, facultative, and anaerobic organisms including* Escherichia coli*, aerobic streptococci,* Bacteroides fragilis, Prevotella,* and anaerobic* Peptostreptococcus*. It is thought that normal vaginal flora or a sexually transmitted organism invades the fallopian tube epithelium, resulting in tissue damage and necrosis. This area of ischemia provides an environment for anaerobes to flourish. The bacteria produce an inflammatory response that spreads outside the fallopian tubes to the other adnexal structures. The body attempts to localize the inflammatory process by enclosing the abscess [1]. The abscess may cause abdominal or pelvic pain, fevers and chills, vaginal discharge, or change in bowel habits [1, 4]. Laboratory studies are nonspecific but may include a leukocytosis, elevated erythrocyte sedimentation rate, or elevated C-reactive protein [4]. Broad-spectrum antibiotics are effective in clearing 70% of TOAs. If patients do not respond to medical management within 48–72 hours then drainage or surgical procedures (hysterectomy or oophorectomy) should be considered [1].

Case reports have shown associations between patients with IUDs and unilateral TOAs [8]. Women infected with HIV are at increased risk of developing a TOA and are more likely to have a complicated clinical course compared with those uninfected with HIV. There is no data supporting early surgical intervention for HIV-infected women with a TOA [9].

US is the primary imaging modality for evaluating patients with suspected TOA. On pelvic ultrasound, TOAs appear as adnexal or retrouterine masses that may be cystic, solid, or complex in nature. Additionally, there may be septations, free fluid in the cul-de-sac, or indistinct uterine margins [10]. Ultrasound accurately identifies a mass in almost all of surgically confirmed TOAs [4].

The most common appearance of TOAs on CT is tubular or spherical cystic adnexal masses with internal septations, relatively uniform wall thickening, and loss of fat planes between the mass and adjacent organs [11]. The presence of internal gas bubbles is the most specific radiologic finding suggestive of TOA, but this is rarely seen [10].

Medical management with broad-spectrum antibiotic therapy is effective in resolving approximately 70% of all TOA cases. One study showed a 72% success rate in patients treated with clindamycin and an aminoglycoside (*n* = 101) compared to an 82% success rate in patients treated with cefoxitin or cefotetan and doxycycline (*n* = 62), but the differences were not statistically significant [1]. Other published studies have also supported the safety and use of these two antibiotic regimens in treatment of TOAs.

Penicillin-based antibiotic therapy is less efficacious in the treatment of TOAs compared with alternative regimens. One study showed only a 42% response rate in patients with TOAs treated with penicillin and aminoglycoside [12]. Ampicillin, which was used in our patient, occasionally is added to antibiotic regimens containing aminoglycoside and either clindamycin or metronidazole to provide sufficient coverage against enterococci. The use of metronidazole, which has bactericidal activity against anaerobes, is less studied in the management of TOAs. If patients do not respond to medical management within 48–72 hours, drainage or surgical procedures (such as hysterectomy or oophorectomy) should be considered [1].

Interventional radiology in the management of TOAs is increasingly being used. A retrospective study in California showed a steady increase in the proportion of hospitalizations associated with drainage procedures, with an increase from 2.6% in 1991 to 7.6% in 2001 [13]. In one study, a combination of antibiotic therapy and ultrasound-guided percutaneous drainage of TOAs was found to be successful in 95% of patients [14]. Transvaginal drainage of TOAs has been shown to be effective with limited complications. The use of either percutaneous or transvaginal drainage of TOAs decreases the need for surgical intervention and prevents associated surgical complications.

Some studies have demonstrated that more aggressive treatment with early drainage of TOAs can improve patient outcome and decrease morbidity, length of hospital stay, and cost. A randomized controlled study of 40 patients by Perez-Medina et al. found that 90% of patients who received a combination of antibiotics and early transvaginal drainage of an unruptured TOA responded successfully to treatment. In contrast, 65% of patients in the control group who received only antibiotic treatment had a favorable outcome [15]. A retrospective study of 302 cases by Gjelland et al. looked at patients who underwent combined antibiotic treatment and transvaginal drainage of a TOA. It found that 93% of the patients did not need surgery or have major complications [16]. While the drainage of TOAs can be effective, up to 25% of all patients with a TOA may still require surgical intervention [1].

A TOA's size helps dictate management and clinical care. A retrospective study by DeWitt et al. found that TOAs with a maximum diameter greater than 8 cm were associated with a higher risk of complications, including increased need for drainage or surgery when compared to smaller abscesses (35% versus 9%, resp., *p* < 0.01) [17]. The study showed a 43% failure rate when only medical management was used for abscesses >8 cm [17]. A study by Reed et al. showed a 35% failure rate for abscesses between 7 and 9 cm and a 60% failure rate for abscesses ≥10 cm [3]. Every 1 cm increase in abscess size has been associated with an increase in hospital stay by 0.4 days per average length of hospitalization of 4.9 ± 1.6 days [17]. There was no difference between the average lengths of hospital stay in patients with TOAs who required readmission within ninety days when compared to those who were not readmitted [13].

We report a complicated case of a TOA requiring percutaneous drainage after failing conservative management with antibiotics. The patient suffered a ruptured spleen leading to hemorrhagic shock and splenectomy and salpingooophorectomy.
